# Assessment of Albumin ECM Accumulation and Inflammation as Novel In Vivo Diagnostic Targets for Multi-Target MR Imaging

**DOI:** 10.3390/biology10100964

**Published:** 2021-09-27

**Authors:** Jana Möckel, Julia Brangsch, Carolin Reimann, Jan O. Kaufmann, Ingolf Sack, Dilyana B. Mangarova, Avan Kader, Matthias Taupitz, Lisa C. Adams, Sarah Keller, Antje Ludwig, Bernd Hamm, Rene M. Botnar, Marcus R. Makowski

**Affiliations:** 1 Department of Radiology, Charité-Universitätsmedizin Berlin, Corporate Member of Freie Universität Berlin and Humboldt-Universität zu Berlin, Charitéplatz 1, 10117 Berlin, Germany; julia.brangsch@charite.de (J.B.); carolinreimann1990@web.de (C.R.); ingolf.sack@charite.de (I.S.); dilyana.mangarova@charite.de (D.B.M.); avan.kader@charite.de (A.K.); matthias.taupitz@charite.de (M.T.); lisa.adams@charite.de (L.C.A.); sarah.keller@charite.de (S.K.); antje.ludwig@charite.de (A.L.); bernd.hamm@charite.de (B.H.); marcus.makowski@tum.de (M.R.M.); 2 Animal Behavior and Laboratory Animal Science, Department of Veterinary Medicine, Institute of Animal Welfare, Freie Universität Berlin, Königsweg 67, Building 21, 14163 Berlin, Germany; 3 Division 1.5 Protein Analysis, Federal Institute for Materials Research and Testing, Richard-Willstätter-Str. 11, 12489 Berlin, Germany; jan-ole.kaufmann@charite.de; 4 Department of Chemistry, Humboldt-Universität zu Berlin, Brook-Taylor-Str. 2, 12489 Berlin, Germany; 5 Department of Veterinary Medicine, Institute of Veterinary Pathology, Freie Universität Berlin, Robert-von-Ostertag-Str. 15, Building 12, 14163 Berlin, Germany; 6 Berlin Institute of Health, Charité-Universitätsmedizin Berlin, Charitéplatz 1, 10117 Berlin, Germany; 7 Medizinische Klinik für Kardiologie und Angiologie, Charité-Universitätsmedizin Berlin, Corporate Member of Freie Universität Berlin and Humboldt-Universität zu Berlin, Campus Mitte, Charitéplatz 1, 10117 Berlin, Germany; 8 DZHK (German Centre for Cardiovascular Research), Partner Site Berlin, Charitéplatz 1, 10117 Berlin, Germany; 9 Wellcome Trust/EPSRC Centre for Medical Engineering, King’s College London, London WC2R 2LS, UK; rene.botnar@kcl.ac.uk; 10 BHF Centre of Excellence, King’s College London, London WC2R 2LS, UK; 11 School of Biomedical Engineering and Imaging Sciences, King’s College London, St Thomas’ Hospital Westminster Bridge Road, London SE1 7EH, UK; 12 Escuela de Ingeniería, Pontificia Universidad Católica de Chile, Santiago 8970117, Chile; 13 Department of Diagnostic and Interventional Radiology, School of Medicine and Klinikum Rechts der Isar, Technical University of Munich, Munich (TUM), Ismaninger Str. 22, 81675 München, Germany

**Keywords:** serum albumin, extracellular matrix, macrophages, atherosclerosis, magnetic resonance imaging

## Abstract

**Simple Summary:**

Atherosclerosis is an inflammatory disease associated with extracellular matrix remodeling. It is characterized by endothelial dysfunction with albumin influx into the vessel wall and macrophage accumulation in atherosclerotic lesions. Non-invasive magnetic resonance imaging (MRI) allows for the assessment of the molecular components of the plaque and vessel wall by using two different target-specific MRI contrast agents in one imaging session. Therefore, multi-target MRI is a promising method to improve diagnosis and treatment monitoring in patients with atherosclerosis.

**Abstract:**

Atherosclerosis is a progressive inflammatory vascular disease characterized by endothelial dysfunction and plaque burden. Extracellular matrix (ECM)-associated plasma proteins play an important role in disease development. Our magnetic resonance imaging (MRI) study investigates the feasibility of using two different molecular MRI probes for the simultaneous assessment of ECM-associated intraplaque albumin deposits caused by endothelial damage and progressive inflammation in atherosclerosis. Male apolipoprotein E-deficient (*ApoE^-/-^*)-mice were fed a high-fat diet (HFD) for 2 or 4 months. Another *ApoE^-/-^*-group was treated with pravastatin and received a HFD for 4 months. T1- and T2*-weighted MRI was performed before and after albumin-specific MRI probe (gadofosveset) administration and a macrophage-specific contrast agent (ferumoxytol). Thereafter, laser ablation inductively coupled plasma mass spectrometry and histology were performed. With advancing atherosclerosis, albumin-based MRI signal enhancement and ferumoxytol-induced signal loss areas in T2*-weighted MRI increased. Significant correlations between contrast-to-noise-ratio (CNR) post-gadofosveset and albumin stain (R^2^ = 0.78, *p* < 0.05), and signal loss areas in T2*-weighted MRI with Perls’ Prussian blue stain (R^2^ = 0.83, *p* < 0.05) were observed. No interference of ferumoxytol with gadofosveset enhancement was detectable. Pravastatin led to decreased inflammation and intraplaque albumin. Multi-target MRI combining ferumoxytol and gadofosveset is a promising method to improve diagnosis and treatment monitoring in atherosclerosis.

## 1. Introduction

Atherosclerosis is a significant cause of morbidity and mortality worldwide [1]. This vascular disease arises from low-density lipoprotein (LDL) influx into the vessel wall’s intimal layer leading to fatty streaks due to endothelial damage and vascular inflammation from the aorta to the coronary arteries [2,3,4]. Extracellular matrix (ECM) remodeling plays a crucial role in developing atherosclerotic lesions and determines the risk of rupture [5,6]. Most notably, atherosclerosis development and progression are accompanied by ECM degradation initiated by matrix metalloproteinases (MMPs) and new ECM protein synthesis induced by proliferating and migrating smooth muscle cells (SMCs) [4]. As the main components of the ECM, collagen I and III and elastin ensure vessel wall stability and integrity of the fibrous cap of an atherosclerotic plaque [7]. Dynamic ECM changes occurring during atherogenesis alter the stability of the vessel wall; these changes are regulated by endothelial cells [7]. In the vessel wall, intense inflammatory processes increase the levels of inflammatory cytokines, which activate macrophages and endothelial cells. Inflammatory processes induce plaque instability and thinning of the fibrous cap due to macrophages and SMCs releasing MMPs that degrade ECM components [7]. These pathological changes have been demonstrated in patients with acute myocardial infarction (MI) and also occur in patients with atherosclerosis [7]. Endothelial dysfunction is a critical atherogenic factor [8]. Vascular permeability is increased in particular due to damaged endothelial glycocalyx, which normally functions as a barrier between the blood and vessel wall [9,10]. As a plasma protein, albumin is bound within the glycocalyx and maintains endothelial stability and integrity [9]. During angiogenesis, plaque neovascularization also leads to enhanced degradation of the ECM [11]. Consequently, blood cells are able to infiltrate the vessel wall, potentially increasing the risk for plaque rupture [10]. Different biochemical processes have already been discovered as potential risk factors for disease onset, such as high cholesterol level, oxidation of low-density lipoproteins (Ox-LDL), inflammatory processes, and hypertension. As the disease progresses, atherosclerotic plaques develop and increase over time [3]. Cardiovascular events, such as a MI or stroke are potentially fatal consequences [12]. Common diagnostic imaging tools to assess atherosclerotic lesions are angiography and intravascular ultrasound [2]. Both interventional techniques are invasive and allow for the detection of arterial stenosis, as well as visualization of plaque and the vessel wall [13], but do not provide information on plaque composition [13,14]. Magnetic resonance imaging (MRI) is a favorable alternative with several advantages: it does not use ionizing radiation and saves the body from invasive interventions [15]. Apart from repeatability and high spatial resolution for vessel wall evaluation [8], imaging in three dimensions is another advantage of MRI [2,15]. Molecular MRI enables visualization of different molecular targets or biomarkers in vivo [2,12] using imaging probes often based on paramagnetic gadolinium or iron oxide particles [12]. As for potential biomarkers for atherosclerosis, serum albumin is used as a target protein [1]. As a plasma component, it only seeps into the damaged vessel wall along several intra- and intercellular pathways when vascular tone becomes imbalanced [10]. In atherosclerotic plaques, albumin is the most abundant plasma protein [16]. During disease progression, albumin infiltrates into the intima and deposits in the adventitia [11], which correlates with increased inflammation [17]. It has been shown that albumin is associated with collagen fibers and elastin microfibrils of the ECM and in the necrotic region of the plaque [5]; therefore, albumin is a promising target for molecular MRI contrast agents [1]. Such contrast-enhancing agents exploit the increased permeability of proliferating vasculature in atherosclerotic plaques [1]. Here, we use gadofosveset as a reversible serum albumin-binding blood pool agent [1,8]. Gadofosveset is a predominantly intravascular agent [8]. Albumin-bound gadofosveset passively enters the plaque and vessel wall through leaky vascular endothelium and permeable neovascular tissues [1,8].

Further MRI probes used for effective visualization of atherosclerotic plaques are ultrasmall superparamagnetic iron oxide (USPIO) particles [18]. USPIOs are used to detect macrophages involved in vessel wall inflammation. Twenty-four hours after intravenous administration, iron oxide particles are taken up by macrophages, causing a signal loss in T2*-weighted MRI. Ferumoxytol is an emerging MRI contrast agent that is used to analyze atherosclerotic pathobiology [18].

Several recent studies have shown the feasibility of the simultaneous use of different contrast agents to improve the characterization of various cardiovascular diseases, including atherosclerosis and abdominal aortic aneurysms [19,20].

The aim of our study is to investigate the feasibility of multi-target MRI using an albumin-specific Gd-based contrast agent and an iron oxide-based macrophage-specific MRI probe to assess and characterize atherosclerotic plaques in a mouse model.

## 2. Materials and Methods

### 2.1. Animals

This animal study was authorized by the Regional Office for Health and Social Affairs Berlin (LAGeSo, G 0143/16, 2016) and conducted in accordance with the local guidelines and provisions for the implementation of the Animal Welfare Act as well as the regulations of the Federation of Laboratory Animal Science Associations (FELASA). The animals used were homozygous apolipoprotein-E-deficient (*ApoE^-/-^*) male mice (*n* = 31) (Forschungseinrichtung für experimentelle Medizin (FEM), Berlin, Germany). Murine models, such as the *ApoE^-/-^*-mouse model, are the most widely used animal models in studies investigating atherogenesis [21]. The distribution of lesion sites is comparable to that in human atherosclerosis [21]. Lesions in *ApoE^-/-^*-mice develop in the aortic root, brachiocephalic artery (innominate artery), and further arterial branches with disturbed blood flow and relatively low shear stress [21]. With regard to sex, men have a higher prevalence of hypertension and a higher cardiovascular risk than women [22]. Moreover, men were found to have a higher total plaque burden and more severe inflammation than women [22]; for this reason, we used male *ApoE^-/-^*-mice.

**Figure 1:**
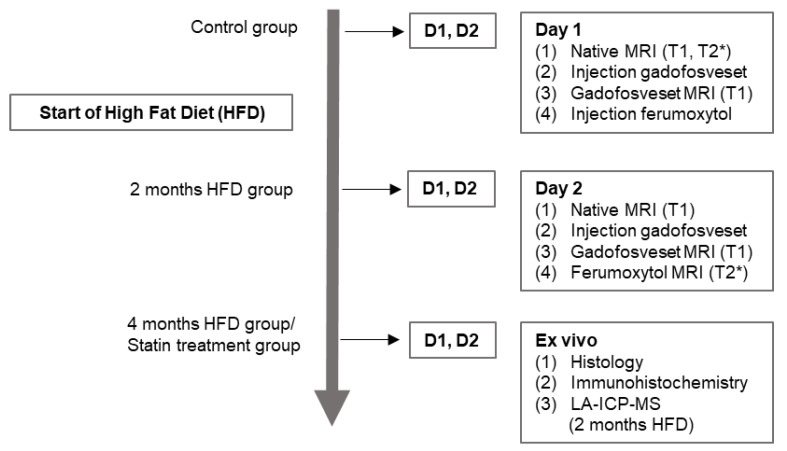
Overview of the experimental design. On day one, the mice underwent a pre-contrast MRI examination including T1- and T2*-weighted sequences. After injection of gadofosveset at a clinical dose of 0.03 mmol/kg, a T1-weighted MRI scan was performed. Subsequently, ferumoxytol was administered at a dose of 4 mg Fe/kg. On day two, an unenhanced (native) T1-weighted MRI sequence was acquired to prove the absence of any residues of gadofosveset. After the second injection of gadofosveset, T1-weighted and subsequent T2*-weighted MRI sequences were acquired. Thereafter, different ex vivo examinations of the brachiocephalic tissue were performed.

With regard to animal housing, the mice were kept in a clean barrier at a constant room temperature of 22 ± 2 °C under 12:12 light/dark cycle conditions. Food and water were supplied ad libitum. Starting at the age of 8 weeks, 31 mice were fed a high-fat diet (HFD) containing 21% lard with 0.15% (*wt*/*wt*) cholesterol (Special-Diets-Services, Witham, UK). Among these 31 *ApoE^-/-^*-mice, 11 mice were fed with HFD for 2 months (*n* = 11), 10 mice were fed with HFD for 4 months (*n* = 10), and 10 *ApoE^-/-^*-mice (*n* = 10) underwent pravastatin (Kemprotec-Limited, Middlesbrough, UK) therapy while being fed the HFD for 4 months. Pravastatin was dissolved and administered via drinking water at a dose of 40 mg/kg body weight per day, which is the standard regimen for treating coronary disease [23,24]. Additionally, a control group, consisting of 26-week-old male C57BL/6J-mice (*n* = 10) purchased from Charles River Laboratories (Sulzfeld, Germany) received a standard lab diet. All animal treatments and medical supplies conformed to our laboratory’s standard protocol [20]. Details of the experimental design are provided in Figure 1.

### 2.2. Gadofosveset Trisodium as a Gd-Based Albumin-Specific Contrast Agent

We used Vasovist^®^ (gadofosveset trisodium, Bayer Healthcare, Inc., Wayne, NJ, USA) as a Gd-based albumin-specific MRI contrast agent at a clinical dose of 0.03 mmol/kg body weight. Gadofosveset trisodium is a blood pool agent. Albumin-bound gadofosveset trisodium has a 5- to 10-fold higher relaxivity (r1 = 18–20 mmol^−1^ s^−1^) than Gd-DTPA (Magnevist, r1 = 3.9 mmol^−1^ s^−1^) or Gd-DOTA (gadobutrol, r1 = 4.7 mmol^−1^ s^−1^) at 1.5 Tesla (T) [25]. Albumin binding is reversible. Even in unbound form, gadofosveset demonstrates a higher relaxivity than gadobutrol and Gd-DTPA because of its increased molecular weight [25].

### 2.3. Iron Oxide Particles as a Macrophage-Specific MRI Probe

Iron oxide particles are used to detect intraplaque macrophage accumulation based on shortening T2/T2* relaxation times. T2*-weighted MR images show a significant signal loss in the vessel wall [26]. Ferumoxytol (Feraheme^®^, AMAG-Pharmaceuticals, Waltham, MA, USA) is used as an off-label MRI contrast agent. Its intravascular half-life is 10–14 h [27] and it is sequestered via the reticuloendothelial system. Moreover, R2 (ferumoxytol in plasma: 65.2 ± 1.8 mmol^−1^ s^−1^) and R2* (ferumoxytol in plasma: 55.7 ± 4.4 mmol^−1^ s^−1^) relaxivities have been determined at 3 T [27]. For our investigations, a clinical dose of 4 mg Fe/kg was administered intravenously.

### 2.4. In Vivo MRI

#### 2.4.1. Mouse MRI Protocol

Mice were fed a HFD for 2 and 4 months before they were anesthetized for the first MRI day. The mice in the control group were 26 weeks old when the MRI was performed. Before starting the MRI, mice were anesthetized by an intraperitoneal injection of medetomidine (500 µg/kg), midazolam (5 mg/kg), and fentanyl (50 µg/kg). The MRI contrast agents were intravenously applied by inserting a needle into the tail vein. The needle was attached to a tube with an inner diameter of 0.28 mm, through which the contrast agents were injected. To keep the body temperature stable during MRI sessions, an MRI-compatible heating system (Model 1025, SA Instruments Inc., Stony Brook, New York, NY, USA) was used. Heart rate and respiration were monitored continuously. For further imaging sessions, the mice were treated with an antagonistic combination of flumazenil (500 µg/kg), atipamezole (2.5 mg/kg), and naloxone (1200 µg/kg). All animal treatments and medical supplies are part of our laboratory’s standard protocol [20]. First, all groups were examined with an unenhanced (native) T1- and T2*-weighted MRI sequences followed by gadofosveset administration via the tail vein. Thirty minutes later, a T1-weighted MRI was performed. Afterward, ferumoxytol was injected intravenously through the tail vein, immediately followed by intraperitoneal administration of the antagonist. On day 2 (24 h later), the mice were anesthetized again and a T1-weighted MRI was performed following gadofosveset administration. Thirty minutes later, a T1-weighted MRI was performed followed by a T2*-weighted sequence. Finally, the animals were euthanized under anesthesia via cervical dislocation to remove the carotid arteries with the brachiocephalic artery and a part of the aorta.

#### 2.4.2. Instrumental MRI Setup

All MRI procedures were performed according to the standard protocol of our laboratory [20]. Mice were examined in a clinical 3T Siemens MRI device (Biograph mMR, Siemens Healthcare, Erlangen, Germany). A heating system appropriate for MRI studies (Model 1025, SA Instruments Inc, Stony Brook, NY, USA) was used for monitoring the body temperature (37 °C) of the mouse during imaging.

#### 2.4.3. Assessment of T1-Weighted MRI with Gadofosveset

First, the carotid arteries, the aorta, and the brachiocephalic artery were identified using a defined low-resolution 3D-localizer sequence in a coronal, sagittal, and transverse orientation. Imaging parameters were defined with a field of view of 280 mm and the number of slices was 10 with a slice thickness of 3 mm; matrix = 320 × 320, TR/TE 7.7/3.7 ms, and the flip angle was 20°. After the scout sequence, a 2D time-of-flight (TOF) sequence of the brachiocephalic artery and the aorta was acquired in the transverse orientation. The TOF sequence was acquired with the following parameters: the matrix was 906 × 906, the FOV was 200 mm, and the number of slices was 26 whereas the slice thickness was set with 500 µm. An in-plane spatial resolution of 0.2 × 0.2 mm was used. TR/TE was defined with 35/4.5 ms as well as a flip angle of 90°. An arterial angiogram of the aortic arch, the brachiocephalic, and the carotid arteries was generated as a maximum intensity projection (MIP) from the TOF sequence. The angiogram was used to plan the subsequent contrast-enhanced sequences. Then, a 2D Look-Locker sequence was performed to determine the blood signal nulling inversion time (TI). After that, the inversion recovery scan could be defined for the acquisition of the gadolinium-based albumin-specific contrast agent series. The parameters of the 2D Look-Locker were specified with a matrix of 750 × 750; the FOV was 300 mm; 0.4 × 0.4 mm of in-plane spatial resolution; the slice thickness was 1.5 mm, the TR which was performed between subsequent IR pulses was defined with 1000 ms; and the flip angle was 15°. A high-resolution 3D inversion recovery gradient echo late gadolinium-enhanced sequence for visualization of the gadolinium-based probe was acquired with the following parameters: a FOV of 57 mm; a 416 × 416 matrix; 370 µm slice thickness; 56 slices; 0.137 × 0.137 mm in-plane spatial resolution; TR/TE, 12.1/5.7 ms; TR of 1000 ms between IR pulses; and a 30° flip angle.

#### 2.4.4. Assessment of T2*-Weighted MRI with Ferumoxytol

The distribution of iron oxide particles in the vascular walls was assessed by a T2*-weighted sequence with the following parameters: a FOV of 150 × 150 mm; 500 µm slice thickness; 32 slices; an 832 × 832 matrix; 0.18 × 0.18 mm in-plane spatial resolution; TR/TE, 17/7.4 ms; Phase Partial Fourier, 6/8; and a 20° flip angle.

#### 2.4.5. Evaluation of T1-Weighted Sequences for Gadofosveset Enhancement

MRI signal intensities were quantified with the OsiriX software (OsiriX Foundation, Geneva, Switzerland, version 5.6). High-resolution images were acquired for morphometric measurement as well as for co-localization of the atherosclerotic plaque as a region of interest (ROI). The highest signal enhancement in the arterial wall was specified as the atherosclerotic plaque. The contrast-to-noise ratio (CNR) was calculated using the following equation: (signal of arterial wall in plaque tissue—blood signal)/noise = CNR. The ROI for noise was represented by the standard deviation of pixel intensity in the background air in front of the brachiocephalic artery.

#### 2.4.6. Evaluation of the T2*-Weighted Sequences for Ferumoxytol

For assessment and quantification of the ferumoxytol-dependent signal loss area [%] in MRI, we compared the pre-contrast T2*-weighted MRI of the vessel wall areas before and 24 h after ferumoxytol injection. The different ROIs were quantified with the same image configuration and 2D composition.

### 2.5. Ex Vivo Examinations

#### 2.5.1. Histology of the Brachiocephalic Artery and Plaque Morphometry

After euthanasia, three mice of each group (*n* = 3/group) were perfused with the MorFFFix^®^ fixative (Morphisto, Frankfurt am Main, Germany) for paraffin embedding of the brachiocephalic arteries. The brachiocephalic tissue harvested from the remaining mice was directly frozen at −80 °C for cryostatic samples. Both types of samples were used for visualization of plaque morphometry and analysis of brachiocephalic tissue. Specifically, paraffin-embedded samples were used for Miller’s Elastica-van-Giesson staining and Perls’ Prussian blue staining and were cut into 9 µm thick serial slices. Elastin-staining was performed for analyzing plaque morphometry whereas Perls’ Prussian blue staining was used for iron oxide particle visualization. For further analysis, histological sections and MRI were co-registered by using reference points (the subclavian artery and the aortic arch). Histological sections were analyzed quantitatively with a microscope (Keyence BZ-X800, Keyence Corporation, Osaka, Japan) in combination with the Keyence software for image analysis of plaque morphometry (BZ-X800 Viewer) and for staining quantification (BZ-X800 Analyzer).

#### 2.5.2. Immunofluorescence Staining

Frozen samples were cut into 9 µm thick slices and incubated with SuperBlock^TM^ blocking buffer (37515, Thermo Fisher Scientific, Dreieich, Germany). After washing with PBS-Tween 20 (pH 7.4, 0.05%), samples with the primary antibodies (CD68 rat anti-mouse, MCA1957GA, Bio-Rad, CA, USA; for albumin: goat polyclonal to mouse, AB19194, Abcam, Cambridge, UK, 1:100) were incubated overnight at +4 °C. After washing, the secondary antibodies (CD68: goat anti-rat IgG H+L, Alexa Flour 568, A11077, Invitrogen from Thermo Fisher Scientific, Germany, 1:200; albumin: donkey anti-goat IgG H&L, Alexa Fluor 568, AB175474, Abcam, Cambridge, UK, 1:500) were applied for one hour at room temperature. Paraffin samples were cut into 9 µm thick serial sections, then deparaffinized and boiled with citrate buffer (pH 6, 0.01 M). The first antibody against CD68 was rabbit anti-mouse polyclonal antibody to CD68 (ab125212, Abcam, Cambridge, UK, 1:100). The corresponding second antibody was donkey anti-rabbit IgG Alexa Fluor 568 (A10042, Thermo Fisher Scientific GmbH, Dreieich, Germany, 1:200). All antibodies were diluted in Dako REAL™ Antibody Diluent (Dako, Denmark). For counterstaining and fixation, all samples were mounted with DAPI Staining Solution (ROTI^®^ Mount FluorCare DAPI, Carl Roth GmbH & Co. KG, Karlsruhe, Germany).

To analyze plaque morphometry and immunofluorescence and histological staining in atherosclerotic plaques, a microscope (Keyence BZ-X800, Keyence Corporation, Osaka, Japan) with image analysis software (BZ-X800 Viewer and BZ-X800 Analyzer, Keyence Corporation, Osaka, Japan) was used.

#### 2.5.3. Gadolinium Localization Using Laser Ablation Inductively Coupled Plasma Mass Spectrometry (LA-ICP-MS)

The Forschungszentrum Jülich (Jülich, Germany) conducted all technical processing and final visualization by a company-owned software. Cryostatic sections of the brachiocephalic arteries that were 9 µm thick were generated. They were collected on SuperFrost Plus adhesion slides at −20 °C (Thermo Fisher Scientific, Dreieich, Germany). Afterwards, the samples were processed with the laser ablation system NWR 213 (New Wave Research, Fremont, CA, USA). It was linked to an inductively coupled plasma mass spectrometer (Agilent 7900, Agilent Technologies, Santa Clara, CA, USA) which performed scans with a measured spot area of 20 µm, a laser speed of 20 µm/s, and an energy level of 34%. Specimens of rat brains with a specified amount of Gd were used as standards and were measured under the same conditions to verify the accuracy of Gd quantification in harvested brachiocephalic arteries.

### 2.6. Statistical Methods

Results are presented as means ± standard deviation values. The Kolmogorov-Smirnov test was used to determine the normal distribution of data. After successful testing for normal distribution, a one-way ANOVA was performed to compare the results between the groups. Afterwards, the Scheffé-post-hoc test was applied. All statistical analyses were performed using Microsoft^®^ Excel^®^ (Microsoft Office Professional Plus © 2016, Microsoft Corporation, Redmond, WA, USA). Statistical significance was specified as *p* < 0.05.

## 3. Results

### 3.1. Assessment of Vessel Wall Inflammation and Permeability by Multi-Target MRI

**Figure 2:**
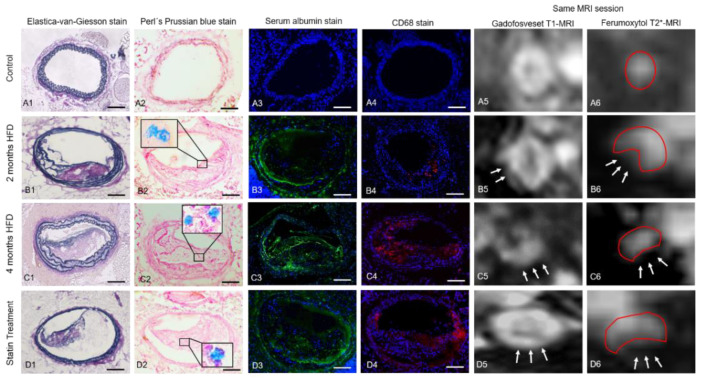
MRI and histological analysis of brachiocephalic tissue. (**A1**–**D4**): Histologically stained cross-sectional slices of the brachiocephalic artery, (**A1**–**D1**) Elastica-van-Giesson stain, (**A2**–**D2**) Perls’ Prussian blue stain, (**A3**–**D3**) immunofluorescence serum albumin stain, (**A4**–**D4**) immunofluorescence CD68 stain of control mice and *ApoE^-/-^*-mice fed with HFD for 2 and 4 months as well as a statin treatment group. The scale displays 100 µm. (**B2**), (**C2**), (**D2**) The enlarged details show iron oxide particle depositions in the plaque. (**A5**–**D5**) and (**A6**–**D6**) T1- and T2*-weighted images corresponding to the histological sections are shown in the first four columns. (**A5**–**D5**) Gadofosveset enhances T1 signal intensity in areas of serum albumin deposition within atherosclerotic plaques. White arrows point to signal-enhanced plaque sites. (**A6**–**D6**) Accumulation of ferumoxytol causes clear areas of signal loss (white arrows) in T2*-weighted images. Signal loss in plaque areas is most conspicuous in the 4 months HFD group. The control group did not show any signal extinction. Red outlines represent the vessel wall delimitation to the extravascular space and the plaque area (signal cancellation in T2*-weighted MRI).

An overview of MRI and histological results are visualized in Figure 2.

**Figure 3:**
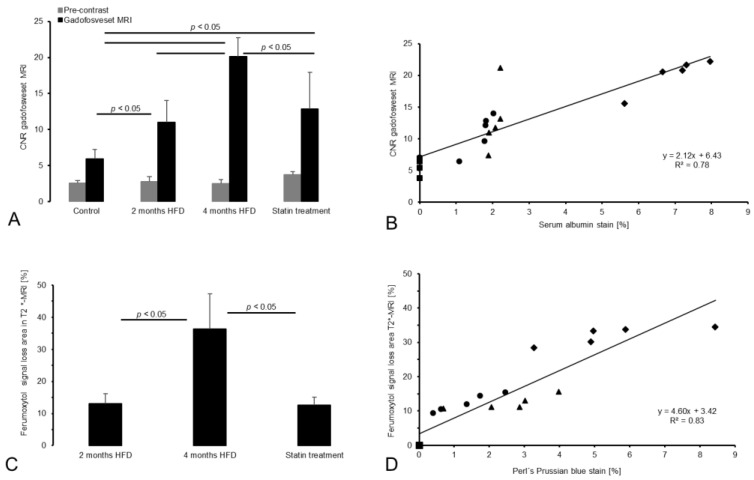
MRI findings following gadofosveset and ferumoxytol administration correlated with histology. (**A**) MRI of the atherosclerotic plaque area before and after administration of gadofosveset. In each group (*n* = 5/group), the signal increased significantly between pre- and post-contrast images (*p* < 0.05). Comparison between groups reveals an increase in CNR with disease progression (*p* < 0.05). (**B**) The scatter plot data show a strong correlation (*p* < 0.05) between CNR after gadofosveset administration and serum albumin staining [%] of the brachiocephalic tissue samples (*n* = 5/group). (**C**) Ferumoxytol-induced signal extinction in the plaque area was most pronounced in the 4 months HFD group. No significant differences were observed between the 2 months HFD group and the statin treatment group (*n* = 5/group). (**D**) The scatter plot demonstrates a significant correlation (*p* < 0.05) between ferumoxytol-induced MRI signal loss and Perls’ Prussian blue stain [%] (*n* = 5/group). Symbols: square—control group; triangle—statin treatment group; circle—2 months HFD group; diamond—4 months HFD group.

**Figure 4:**
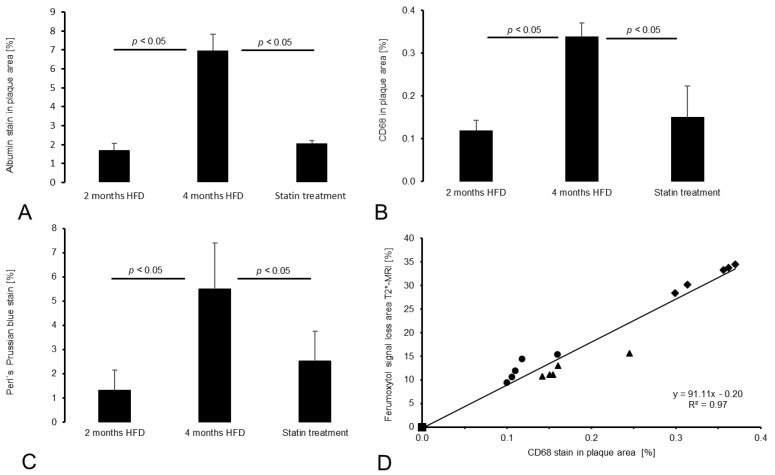
Histological analysis. (**A**) The bar chart shows the averaged fluorescent serum albumin-stained areas [%] within plaques for the three experimental groups (*n* = 5/group). (**B**) Macrophage-specific CD68 staining in the plaque area [%] of the experimental groups is demonstrated (*n* = 5/group). (**A**) and (**B**) Both fluorescent staining methods indicate significant differences in the 2 months HFD group and the statin treatment group compared with the 4 months HFD group (*p* < 0.05). In contrast, the 2 months HFD and the statin treatment group do not differ significantly (*p* > 0.05). (**C**) The Perls’ Prussian blue stain area [%] within plaques of the experimental groups show significant differences in the 4 months HFD group compared with the 2 months HFD group as well as the statin treatment group (*p* < 0.05). In contrast, the 2 months HFD group and the statin treatment group do not differ significantly (*p* > 0.05; *n* = 5/group). (**D**) The scatter plot indicates a strong correlation of CD68 macrophage staining in the plaque area and the T2* signal loss area (*p* < 0.05; *n* = 5/group). Symbols: square—control group; triangle—statin treatment group; circle—2 months HFD group; diamond—4 months HFD group.

No plaque development was observed in the control group. CNR in gadofosveset-enhanced images was lowest in the control group and clearly increased with plaque burden (Figure 3A). Moreover, there was a strong correlation between the albumin-specific signal enhancement and albumin staining (Figure 3B). In addition, both the area of T2* signal loss and the area of CD68 staining increased significantly with disease progression (Figure 3 and Figure 4). In the statin treatment group, MRI signal intensities as well as histological and immunohistochemical findings differed significantly from the results in the untreated 4 months HFD group, consistent with less extensive plaque formation.

### 3.2. Gadofosveset-Enhanced T1-Weighted MRI

In each group, T1 signal enhancement in the brachiocephalic plaque area was significant after the gadofosveset administration compared with pre-contrast images (*p* < 0.05; Figure 3A).

After gadofosveset injection, MRI signal enhancement was highest in the 4 months HFD group. The 2 months HFD group showed lower CNR values than both the untreated 4 months HFD group and the statin treatment group. The control group showed the lowest post-contrast CNR. The post-contrast CNR in the control group differed significantly from that of the 2 months HFD group (*p* < 0.05) and 4 months HFD group (*p* < 0.05). Significant differences were also present between the 4 months HFD group and statin treatment group (*p* < 0.05). No significant difference was observed between the 2 months HFD and statin treatment group (*p* > 0.05).

### 3.3. T2*-Weighted MRI for Assessment of Inflammatory Processes

Before ferumoxytol administration, the brachiocephalic vessel wall was clearly circularly shaped in control mice on T2*-weighted sequences (Figure 2). Twenty-four hours after ferumoxytol injection, areas of MRI signal loss [%] were present in the vessel wall in the 2 months HFD, 4 months HFD, and statin treatment groups (Figure 2). In all three groups, there was a partial loss of the MRI signal in plaque areas in the brachiocephalic artery. The signal loss area increased with plaque progression. The area of signal loss was larger in the 4 months HFD group (36.3 ± 11%; *p* < 0.05) than in the 2 months HFD group (13.2 ± 3%, *p* < 0.05) and the statin treatment group (12.6 ± 2.5%; *p* < 0.05). No significant difference was observed between the 2 months HFD and the statin treatment groups (*p* > 0.05, Figure 3C).

### 3.4. Histology and Immunofluorescence

#### 3.4.1. Intraplaque Albumin as Biomarker for Vessel Wall Permeability

The 4 months HFD group demonstrated a significantly (*p* < 0.05) larger fluorescent albumin-stained area compared to the 2 months HFD group and to the statin treatment group (*p* < 0.05, Figure 4A).

By contrast, the 2 months HFD group and the statin treatment group did not differ significantly from each other (*p* > 0.05). The control group showed no plaque development.

#### 3.4.2. CD68 and Perls’ Prussian Blue Staining for Macrophage and Iron Oxide Particle Detection

CD68-stained areas as well as Perls’ Prussian blue-stained area [%] within plaques increased significantly (*p* < 0.05, Figure 4) with HFD duration. Specifically, the 4 months HFD group presented the largest CD68 area [%] in comparison to the 2 months HFD (*p* < 0.05) or statin treatment group (*p* < 0.05) while no significant difference was observed between the 2 months HFD and statin treatment groups (*p* > 0.05). Similar results were obtained with Perls’ Prussian blue staining, see Figure 3C. There was a strong correlation between T2* signal loss [%] and CD68 staining (y = 91.11x − 0.20, R^2^ = 0.97, Figure 4D).

#### 3.4.3. Influence of Ferumoxytol on Gadofosveset Signal Enhancement

**Figure 5:**
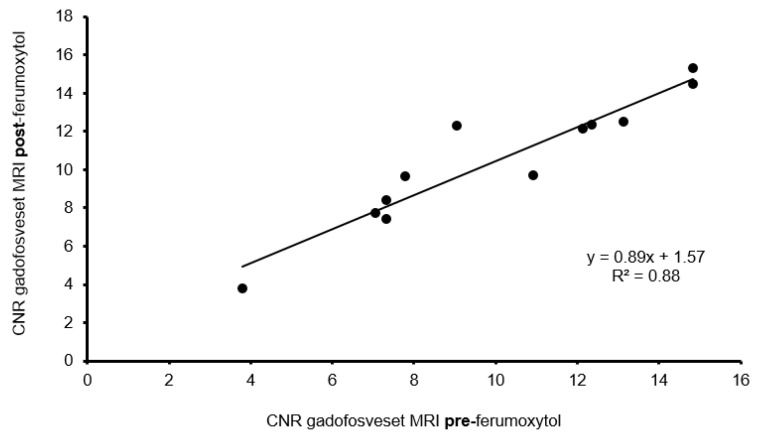
Ferumoxytol impact on T1-weighted MRI with gadofosveset. T1-weighted MRI using serum albumin contrast agent was performed before and 24 h after iron oxide particle administration (*n* = 3/group). The pronounced correlation of CNR pre- and post-iron oxide particle administration rules out a negative influence on the serum albumin probe.

To rule out interference between the two contrast agents used in our experiment, we investigated the possible effects of ferumoxytol on gadofosveset enhancement in three mice from each group (*n* = 3/group). The analysis was performed using the T1-weighted sequences from day one before ferumoxytol injection and from day two, i.e., 24 h after ferumoxytol administration and after repeated gadofosveset administration. The CNR values of the Gd-based albumin probe showed a strong correlation between both MRI sessions (y = 0.89x + 1.57, R^2^ = 0.88, Figure 5). There was no significant difference in signal enhancement (*p* > 0.05).

### 3.5. Correlation between In Vivo and Ex Vivo Analysis

CNR after administration of the albumin-specific probe and extent of the fluorescent albumin staining [%] in the plaque area showed strong correlation (y = 2.12x + 6.43, R^2^ = 0.78, *p* < 0.05; see Figure 3B). Moreover, there was a strong correlation between the areas of T2* signal loss and plaque areas positive for Perl s’ Prussian blue staining (y = 4.60x + 3.42, R^2^ = 0.83, *p* < 0.05, see Figure 3D).

### 3.6. LA-ICP-MS for Analysis of Gadolinium Distribution in the Brachiocephalic Artery

**Figure 6:**
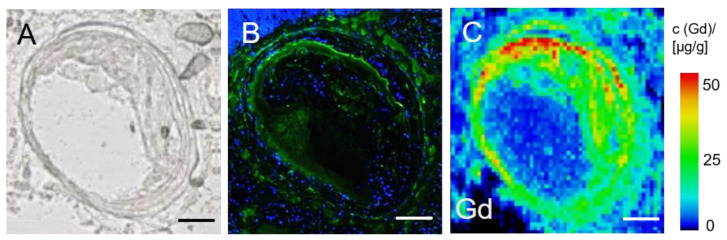
LA-ICP-MS analysis of gadolinium distribution in an atherosclerotic plaque. (**A**) Native cryosection of the brachiocephalic artery of an *ApoE^-/-^*-mouse after 2 months of HFD. (**B**) Serum albumin-specific immunohistological staining of a serial section. (**C**) LA-ICP-MS showing gadolinium distribution across the brachiocephalic wall in a serial section of the same sample and a clear colocaliaztion of Gd and areas positive for albumin staining (**A**). The scale displays 100 µm.

LA-ICP-MS analysis was performed in three mice of the 2 months HFD group (*n* = 3). The vessel wall, specifically the plaque area, showed a clear accumulation of gadolinium from the serum albumin-specific MR probe whereas the vessel lumen and surrounding tissue did not show massive gadolinium deposition (Figure 6). Furthermore, a significant qualitative spatial co-localization of gadolinium with albumin was demonstrated.

## 4. Discussion

In this study, we examined the feasibility of assessing vessel wall dysfunction by monitoring deposits of extracellular matrix-associated intraplaque albumin and progressive inflammation using two different contrast agents in one single MRI session. Simultaneous use of both contrast agents—ferumoxytol and gadofosveset—seems to be a promising method to determine vessel wall permeability and inflammatory processes in the context of progressive atherosclerosis. Furthermore, we studied the response of disease progression to statin treatment.

Serum albumin has been used as a surrogate marker for endothelial damage [28] because it is the most abundant plasma protein in atherosclerotic lesions [16]. A major underlying mechanism is the higher permeability of atherosclerotic lesions [28]. In our study, the amount of intraplaque albumin increased significantly with disease progression (Figure 3 and Figure 4), and we found similar amounts of albumin in the 2 months HFD group and statin treatment group. Mice of the 4 months HFD group showed the highest albumin-specific signal enhancement compared with the 2 months HFD and statin treatment group (Figure 3A). The results of Phinikaridou and colleagues who investigated vessel wall signal enhancement in *ApoE^-/-^*-mice with advanced atherosclerosis and pravastatin treatment are in agreement with our results [8]. Phinikaridou et al. observed a gradually increasing vessel wall permeability and albumin-induced gadosfosveset-based signal enhancement with plaque progression and longer HFD duration [8]. The study of Rademakers et al. also showed albumin influx into the adventitia in aged *ApoE^-/-^*-mice due to a higher density of leaky plaque-associated microvessels and hyperpermeability in advanced lesions [17]. Plaque-associated neovascularization initially occurs in the adventitia at lesion sites and close to the elastic lamina in advanced plaques [17]. Londono and colleagues investigated albumin deposits in human atherosclerotic lesions by using protein-A gold immunocytochemistry [5]. They found albumin deposits at collagen fibers in the ECM of fibrous plaques, in the periphery of necrotic plaque regions, and associated with elastin microfibrils [5]. These results corroborate our investigations because those binding sites correspond to the plaque-related MRI signal enhancement and histological results in our current study (see Figure 2). With regard to previous work our group performed by Adams et al. [19], our MRI measurements show similar results. Adams and colleagues aimed to assess the risk of rupture of abdominal aortic aneurysms (AAA) caused by increased vascular permeability in *ApoE^-/-^*-mice [19]. In fact, Adams et al. achieved the highest CNR values after administration of gadofosveset and histologically assessed extraluminal albumin accumulations in advanced AAA compared to early-stage AAA or control mice [19]. During atherogenesis, further ECM changes occur with respect to arterial wall stiffness and collagen structures. First, arterial walls become stiff because of collagen I and III deposits and cross-linking-processes as well as elastin fragmentation. Vessel wall dysfunction is also associated with greater stiffness [29]. Moreover, collagen contributes to intimal calcification and may increase arterial stiffness [30]. During progressive inflammation, collagen synthesis is increased [31]. Due to these ECM changes during progressive atherogenesis, the increased albumin influx and depositions in the lesion sites may be associated with the enhanced collagen synthesis and the progressive arterial wall stiffening.

With regard to possible interactions between intraplaque albumin and ECM, Stephan et al. used cultured human renal proximal tubular epithelial cells to investigate the influences of albumin on extracellular components [32]. Stephan and colleagues found that albumin stimulates the accumulation of ECM proteins by upregulating the levels of tissue inhibitors of metalloproteinases (TIMP) 1 and 2, thereby inhibiting the breakdown of ECM proteins [32]. These findings suggest that further investigation of possible interactions between albumin and ECM components, which are not yet fully elucidated in the context of atherosclerosis, might be promising.

Statins have a plasma lipid-lowering function as well as non-lipid lowering transcriptional influence, especially anti-inflammatory effects during atherogenesis and also on endothelial cells [33]. Statins suppress the expression of endothelial adhesion molecules and cytokines and reduce macrophage infiltration in the early stages of atheroma [33]. These effects explain our finding that the statin treatment group had lower amounts of albumin and macrophages in the plaque compared to the untreated 4 months HFD group. We observed a significant correlation between albumin-specific immunohistochemistry and MRI signal enhancement (Figure 3B). The CNR post-gadofosveset injection was significantly higher in our experimental groups compared to the control group. Approximately 33% of albumin is present in the vascular system whereas ca. 67% can be found in the extravascular area [25]. It has also been shown, that 60% of gadofosveset binds to albumin whereas 40% remains as an unbound fraction when incubated with murine plasma [25]. In fact, gadofosveset could have bound to albumin intra- and extravascularly because of leaky neovessels and endothelial injuries [25]. That given, the signal amplification is significantly higher in the 2 months HFD, 4 months HFD, and statin treatment groups than in the control group. Nevertheless, the control group that consisted of C57BL/6J-mice fed a standard lab diet showed some signal enhancement although no plaque formation is detectable. Here, the vessel wall of the brachiocephalic artery seemed to be intact, so gadofosveset could have bound to circulating albumin in the blood stream. Moreover, in healthy vessels, albumin molecules are partly bound within the glycocalyx to the endothelial surface [9] leading to little gadofosveset deposition at the endothelial surface. Between the 4 months HFD, 2 months HFD, and statin treatment group we observed significantly different post-gadofosveset signal enhancement with progressive atherosclerosis. Notably, the 2 months HFD group showed less signal amplification than the 4 months HFD group and the statin treatment group (Figure 3A). The duration of the experimental treatment, especially HFD duration [8], may play a role in continuous albumin probe diffusion into the vessel wall.

Ferumoxytol is commonly used for vascular MRI of the carotid arteries in patients with atheroma [34]. USPIOs are taken up by macrophages that mediate inflammatory processes in atherogenesis [34]. Our results show the highest ferumoxytol uptake in the 4 months HFD group compared to the 2 months HFD or statin treatment group (see Figure 3). These findings suggest that, with plaque progression, more iron oxide particles accumulate in the plaque area and more macrophages are detectable. Our findings correspond reasonably well to the clinical study of Smits et al., who demonstrated the feasibility of using ferumoxytol in an USPIO-enhanced MRI of the carotid arterial wall in patients with carotid stenosis [35]. It has been shown that ferumoxytol is selectively taken up in plaques 72 h after injection but not in healthy vessel walls [35]. This observation may be attributable to leaky vessel wall endothelium and neovessels within the plaques [35]. Ferumoxytol is sensitive to inflammatory processes in atherosclerosis by entering endothelial leakage areas, in particular plaque sites. Additionally, active inflammation increases the risk of plaque rupture, which in turn can cause myocardial infarction or other cardiovascular events [35]. Due to the prolonged R2* relaxation rate [35], T2*-weighted MR imaging is a powerful method to detect inflammatory processes by visualizing areas of USPIO accumulation [36]. As a biomarker for inflammatory cells such as macrophages, CD68 is also capable of detecting foam cells originating from smooth muscle cells (SMCs) [37]. These cholesterol-loaded SMCs have a macrophage-like phenotype with partially similar functions, as demonstrated in the study of Rong et al. [37]. Therefore, CD68 may detect not only macrophages but also other inflammatory cells with similar phenotype and function, such as cholesterol-loaded SMCs. In general, multiple cell types including VSMCs, immune cells, and endothelial cells seem to be involved in atherosclerotic inflammation [38]. While Rademakers et al. demonstrated an increase in adventitial inflammation, mostly driven by macrophages in advanced plaques [17,39], Sorokin et al. [38] found VSMCs to play a key role in inflammation due to their plasticity. Environmental changes (e.g., shear stress, hyperlipidemia, and lipid accumulation) are sensed by VSMCs leading to phenotypic and functional modification [38]. In fact, these new cell features are associated with atherosclerotic hallmarks and lead to the release of several pro-inflammatory cytokines which in turn influence the progression of atherosclerosis [38,40]. With regard to pravastatin treatment, the study of Chen et al. supports our current findings [41]. Chen et al. report that pravastatin reduces macrophages accumulations in lesion areas in 22-week-old *ApoE^-/-^*-mice [41]. Finally, our findings also suggest that ferumoxytol does not seem to impair the efficacy of the serum albumin-specific MR probe. In conclusion, a molecular multi-target MRI proved to be a feasible method to assess vessel wall dysfunction as well as plaque burden and inflammatory processes in a murine model of progressive atherosclerosis. The investigation of extracellular matrix-associated intraplaque albumin and proinflammatory macrophages as atherosclerotic biomarkers might allow better prediction of the risk of plaque rupture and can be used to detect treatment effects.

### 4.1. Limitations

This study has several limitations. First, the use of ferumoxytol was off-label. It is clinically used as a therapeutic iron supplement, especially in patients with chronic kidney disease [42]. Moreover, imaging with two MRI probes remains to be investigated further before its potential use in humans. Up to now, only a few murine studies have investigated the combined use of two MRI probes in one imaging session [19,20].

### 4.2. Translational Study Design

First, the MRI device and the MRI coil we used were standard clinical devices for patients. Gadofosveset is already a standard clinical MRI contrast agent. Ferumoxytol is a clinically approved parenteral drug for iron substitution and was used here as an MRI contrast agent under off-label conditions. Both agents were used at clinical doses.

## 5. Conclusions

A single in vivo molecular MRI session implementing a dual-probe application was shown to be a feasible method to assess vessel wall dysfunction by detecting matrix-associated albumin deposits, as well as plaque burden and inflammatory processes in mice suffering from progressive atherosclerosis. Further investigation of albumin and macrophages as atherosclerotic biomarkers might allow better estimation of the risk for plaque rupture and assessment of treatment responses.

## Data Availability

Data are available from the corresponding author upon reasonable request.

## Abbreviations

AAA	abdominal aortic aneurysm
*ApoE^-/-^*	apolipoprotein E-deficient
CNR	contrast-to-noise-ratio
ECM	extracellular matrix
EvG	Elastica-van-Gieson
FELASA	Federation of Laboratory Animal Science Associations
FEM	Forschungseinrichtung für experimentelle Medizin
FDA	Food and Drug Administration
FOV	field of view
Gd	Gadolinium
HE-	hematoxylin and eosin
HFD	high fat diet
LA-ICP-MS	laser coupled mass spectrometry
LDL	low-density lipoprotein
MI	myocardial infarction
MIP	maximum intensity projection
MR	magnetic resonance
MRI	magnetic resonance imaging
Ox-LDL	oxidized low-density lipoproteins
ROI	region of interest
SMCs	smooth muscle cell
TE	echo time
TI	inversions time
TIMP	tissue inhibitor of metalloproteinase
TOF	time of flight
TR	repetitions time
USPIO	ultrasmall superparamagnetic iron oxide
VSCM	vascular smooth muscle cell
3D	three-dimensional
2D	two-dimensional
